# Differential regulation of adipose tissue and vascular inflammatory gene expression by chronic systemic inhibition of NOS in lean and obese rats

**DOI:** 10.1002/phy2.225

**Published:** 2014-02-07

**Authors:** Jaume Padilla, Nathan T. Jenkins, Pamela K. Thorne, Kasey A. Lansford, Nicholas J. Fleming, David S. Bayless, Ryan D. Sheldon, R. Scott Rector, M. Harold Laughlin

**Affiliations:** 1 Nutrition and Exercise Physiology, University of Missouri, Columbia, Missouri; 2 Child Health, University of Missouri, Columbia, Missouri; 3 Dalton Cardiovascular Research Center, University of Missouri, Columbia, Missouri; 4 Kinesiology, University of Georgia, Athens, Georgia; 5 Biomedical Sciences, University of Missouri, Columbia, Missouri; 6 Medical Pharmacology and Physiology, University of Missouri, Columbia, Missouri; 7 Harry S Truman Memorial VA Medical Center, Columbia, Missouri; 8 Internal Medicine‐Division of Gastroenterology and Hepatology, University of Missouri, Columbia, Missouri

**Keywords:** Inflammation, L‐NAME, obesity, vascular function

## Abstract

We tested the hypothesis that a decrease in bioavailability of nitric oxide (NO) would result in increased adipose tissue (AT) inflammation. In particular, we utilized the obese Otsuka Long Evans Tokushima Fatty rat model (*n* = 20) and lean Long Evans Tokushima Otsuka counterparts (*n* = 20) to determine the extent to which chronic inhibition of NO synthase (NOS) with *N*^*ω*^‐nitro‐l‐arginine methyl ester (L‐NAME) treatment (for 4 weeks) upregulates expression of inflammatory genes and markers of immune cell infiltration in retroperitoneal white AT, subscapular brown AT, periaortic AT as well as in its contiguous aorta free of perivascular AT. As expected, relative to lean rats (% body fat = 13.5 ± 0.7), obese rats (% body fat = 27.2 ± 0.8) were hyperlipidemic (total cholesterol 77.0 ± 2.1 vs. 101.0 ± 3.3 mg/dL), hyperleptinemic (5.3 ± 0.9 vs. 191.9 ± 59.9 pg/mL), and insulin‐resistant (higher HOMA IR index [3.9 ± 0.8 vs. 25.2 ± 4.1]). Obese rats also exhibited increased expression of proinflammatory genes in perivascular, visceral, and brown ATs. L‐NAME treatment produced a small but statistically significant decrease in percent body fat (24.6 ± 0.9 vs. 27.2 ± 0.8%) and HOMA IR index (16.9 ± 2.3 vs. 25.2 ± 4.1) in obese rats. Further, contrary to our hypothesis, we found that expression of inflammatory genes in all AT depots examined were generally unaltered with L‐NAME treatment in both lean and obese rats. This was in contrast with the observation that L‐NAME produced a significant upregulation of inflammatory and proatherogenic genes in the aorta. Collectively, these findings suggest that chronic NOS inhibition alters transcriptional regulation of proinflammatory genes to a greater extent in the aortic wall compared to its adjacent perivascular AT, or visceral white and subscapular brown AT depots.

## Abstract (precis)

We tested the hypothesis that a decrease in bioavailability of nitric oxide (NO) would result in increased adipose tissue (AT) inflammation. Our findings suggest that chronic NOS inhibition alters transcriptional regulation of proinflammatory genes to a greater extent in the aortic wall compared to its adjacent perivascular AT, or visceral white and subscapular brown AT depots.

## Introduction

Originally characterized as a mediator of vascular smooth muscle relaxation (Ignarro et al. 1987; Palmer et al. 1987), nitric oxide (NO) has since been implicated in a wide range of physiological processes in different tissues including adipose tissue (AT). For example, a recent study in mice demonstrated that overexpression of endothelial NO synthase (eNOS) prevents diet‐induced obesity and that the mechanism of this antiobesogenic effect of eNOS was related to an increase in mitochondrial abundance and activity in visceral AT (Sansbury et al. 2012). Furthermore, while the anti‐inflammatory effects of NO on the vasculature are established (Rudic et al. 1998; Laroux et al. 2001; Kuhlencordt et al. 2009; Gomez‐Guzman et al. 2011; Hossain et al. 2012), recent evidence indicates that NO also exerts an anti‐inflammatory effect in AT. In this regard, eNOS knockout mice exhibit increased inflammation in epidydimal AT, compared to wild‐type counterparts, indicating that NO derived from eNOS is crucial for maintenance of a low‐inflammatory state within the visceral AT (Handa et al. 2011). Whether the anti‐inflammatory influence of NO signaling is also present in other AT depots beyond the viscera including perivascular and brown AT is uncertain.

Accordingly, we utilized the obese hyperphagic Otsuka Long Evans Tokushima Fatty (OLETF) rat model and lean counterparts (Long Evans Tokushima Otsuka [LETO]) to test the hypothesis that a decrease in NO bioavailability with chronic systemic inhibition of NOS activity would result in AT inflammation as well as to determine whether this effect would be modulated with obesity. Examination of the effects of NOS inhibition in both lean and obese rats is important because obesity is associated with AT inflammation (Wellen and Hotamisligil 2003; Shoelson et al. 2006; Gutierrez et al. 2009) as well as reduced NO bioavailability (Williams et al. 2002; Siervo et al. 2011). A question that remains largely unanswered is whether obesity‐associated low NO bioavailability mediates AT inflammation. To begin addressing this question, in this study, we created a lean condition with induced low NO bioavailability to compare it to an obese condition with inherent low NO bioavailability. We hypothesized that systemic NOS inhibition would make the AT of lean animals phenotypically resemble the AT of obese animals. In particular, expression of inflammatory genes and markers of immune cell infiltration were assessed in retroperitoneal white AT, subscapular brown AT, periaortic AT, and its contiguous aorta free of perivascular AT in lean and obese rats systemically treated or not with NOS inhibitor *N*^*ω*^‐nitro‐l‐arginine methyl ester (L‐NAME). The aorta was also included to determine whether the extent of the effects of systemic NOS inhibition on AT samples were comparable to the effects observed in vascular tissue.

## Methods

### Animals

All animal protocols were approved by the University of Missouri Institutional Animal Care and Use Committee. Male LETO (*n* = 20) and OLETF (*n* = 20) rats (age 4 weeks; Tokushima Research Institute, Otsuka Pharmaceutical, Tokushima, Japan) were individually housed on a 12‐h:12‐h light–dark cycle and provided water and standard rodent chow (Formulab 5008; Purina Mills, St. Louis, MO) ad libitum with ~26% protein, 18% fat, and 56% carbohydrate. Body weights and food intakes were recorded on a weekly basis. At 16 weeks of age rats were randomly divided into L‐NAME‐treated (LETO L‐NAME, *n* = 10; OLETF L‐NAME, *n* = 10) or untreated (LETO CONT, *n* = 10; OLETF CONT, *n* = 10) groups. L‐NAME animals received L‐NAME daily in drinking water for 4 weeks as described (Lloyd et al. 2001). Briefly, L‐NAME was dissolved in tap water and the water was supplied fresh every other day to the animals. The concentration of L‐NAME in water was individually adjusted for bodyweight and water consumption such that each rat consumed 65–70 mg/kg per day. Similar dose of L‐NAME in drinking water has been used in previous studies (Lloyd et al. 2001; Gomez‐Guzman et al. 2011). L‐NAME administration was continued until the day of sacrifice (20 weeks of age). On that morning, rats were anesthetized by intraperitoneal injection of pentobarbital sodium (50 mg/kg) following a 12‐h overnight fast. Tissues were harvested and the animals were euthanized by exsanguination in full compliance with the American Veterinary Medical Association Guidelines on Euthanasia.

### Body composition, blood parameters, and citrate synthase activity

On the day of the experiments, body mass was measured to the nearest 0.01 g and, following anesthetization, body composition was determined using a dual energy x‐ray absorptiometry instrument (Hologic QDR‐1000, Hologic, Inc., Bedford, MA) calibrated for rodents. In addition, retroperitoneal, epididymal, and omental fat pad weights were measured to the nearest 0.01 g. Plasma samples were prepared by centrifugation and stored at −80°C until analysis. Glucose, triglycerides, and nonesterified fatty acids (NEFA) assays were performed by a commercial laboratory (Comparative Clinical Pathology Services, Columbia, MO) on an Olympus AU680 automated chemistry analyzer (Beckman‐Coulter, Brea, CA) using commercially available assays according to manufacturer's guidelines. Plasma insulin concentrations were determined using a commercially available, rat‐specific ELISA (Alpco Diagnostics, Salem, NH). In addition, plasma samples were assayed for concentrations of leptin, monocyte chemotactic protein‐1 (MCP‐1), tumor necrosis factor alpha (TNF‐*α*), and interleukin 6 (IL‐6) using a multiplex cytokine assay (Millipore Milliplex, cat no. RCYTOMAG‐80K; Billerica, MA) on a MAGPIX instrument (Luminex Technologies; Luminex Corp., Austin, TX) according to the manufacturer's instructions (Jenkins et al. 2012; Padilla et al. 2013b). Serum nitrate + nitrite (NOx) levels were measured using the Nitrate/Nitrite Fluorometric Assay Kit (Cayman Chemical, item no. 780051, Ann Arbor, MI) according to manufacturer's instructions. Citrate synthase activity was determined in retroperitoneal AT homogenates using the methods of Srere (Srere 1969). AT was homogenized in buffer (25 mmol/L Tris HCl, 1 mmol/L EDTA, pH 7.4), centrifuged, and the infranatant was collected. The citrate synthase activity assay was only performed in the retroperitoneal fat depot because this is where we had AT availability.

### Tissue sampling

A segment of the thoracic aorta cleaned of perivascular AT and excess adventitia, as well as the perivascular AT surrounding the thoracic aorta, retroperitoneal white AT, and subscapular brown AT were quickly excised from the anesthetized rat. Isolated aortic segments were kept in RNAlater (Ambion, Austin, TX) for 24 h at 4°C, then removed from the RNAlater solution and stored at −80°C until analysis. For each fat depot, a portion was flash frozen and kept at −80°C for examination of gene expression and a portion was fixed in neutral‐buffered 10% formalin for histology analysis. Retroperitoneal AT was studied because it is the largest visceral AT depot in obese rats. Subscapular brown AT was studied because it is a source of healthy AT (Stanford et al. 2013) and a classic depot for the study of brown AT biology (Saha et al. 1997; Becerril et al. 2010; Vasilijevic et al. 2010; Sacks and Symonds 2013). Periaortic AT was selected because increasing amounts of evidence implicates AT surrounding the arteries as a local source of proinflammatory cytokines influencing the athero‐susceptibility of the vascular wall (Mazurek et al. 2003; Cheng et al. 2008; Gorter et al. 2008; Chatterjee et al. 2009; Greif et al. 2009; Payne et al. 2012a,b; Szasz and Webb 2012). On the other hand, like others, we have found that AT surrounding the descending thoracic aorta is a brown‐like AT depot in rodents (Fitzgibbons et al. 2011; Padilla et al. 2013c). Last, we studied gene expression in the aorta to determine the extent to which changes in perivascular AT gene expression induced by NOS inhibition and obesity were comparable to changes in gene expression in the adjacent arterial wall.

### RNA extraction and real‐time PCR

AT and aortic samples were homogenized in TRIzol solution using a tissue homogenizer (TissueLyser LT, Qiagen, Valencia, CA). Total RNA was isolated using the Qiagen's RNeasy Lipid Tissue Kit and assayed using a Nanodrop spectrophotometer (Thermo Scientific, Wilmington, DE) to assess purity and concentration. First‐strand cDNA was synthesized from total RNA using the High Capacity cDNA Reverse Transcription kit (Applied Biosystems, Carlsbad, CA). Quantitative real‐time polymerase chain reaction (PCR) was performed as previously described (Padilla et al. 2013b,c using the CFX Connect Real‐Time PCR Detection System (BioRad, Hercules, CA). Primer sequences (Table 1) were designed using the National Center for Biotechnology Information Primer Design tool. All primers were purchased from IDT (Coralville, IA). A 20‐*μ*L reaction mixture containing 10 *μ*L iTaq UniverSYBR Green SMX (BioRad, Hercules, CA) and the appropriate concentrations of gene‐specific primers plus 4 *μ*L of cDNA template were loaded in each well of a 96‐well plate. All PCR reactions were performed in duplicate. PCR was performed with thermal conditions as follows: 95°C for 10 min, followed by 40 cycles of 95°C for 15 sec, and 60°C for 45 sec. A dissociation melt curve analysis was performed to verify the specificity of the PCR products. 18S primers were used to amplify the endogenous control product. Our group has established that 18S is a suitable house‐keeping gene for real‐time PCR when examining AT gene expression (Jenkins et al. 2013; Padilla et al. 2013c). In the present study, 18S CTs were not different among fat depots or groups of animals. Similarly, 18S CTs in the aorta were not different among groups of animals. mRNA expression values are presented as 2^ΔCT^ whereby ΔCT = 18S cycle threshold (CT) – gene of interest CT (Padilla et al. 2013b,c. AT mRNA levels were normalized to perivascular AT in the LETO control group of rats, which was always set at 1. Similarly, aorta mRNA levels were normalized to the LETO control group of rats, which was set at 1.

**Table 1.** Forward and reverse primer sequences for quantitative real‐time PCR.

Gene	Primer sequence (5′3′)	
	Forward	Reverse
18S	GCCGCTAGAGGTGAAATTCTTG	CATTCTTGGCAAATGCTTTCG
Leptin	GACACCCTTAGAGGGGGCTA	AACCCAAGCCCCTTTGTTCA
MCP‐1	CTGTCTCAGCCAGATGCAGTTAA	AGCCGACTCATTGGGATCAT
TNF‐*α*	AACACACGAGACGCTGAAGT	TCCAGTGAGTTCCGAAAGCC
IL‐6	AGAGACTTCCAGCCAGTTGC	AGCCTCCGACTTGTGAAGTG
IL‐10	CTGGCTCAGCACTGCTATGT	GCAGTTATTGTCACCCCGGA
IL‐18	ACAGCCAACGAATCCCAGAC	ATAGGGTCACAGCCAGTCCT
E‐Selectin	GCCATGTGGTTGAATGTAAAGC	GGATTTGAGGAACATTTCCTGACT
VCAM‐1	GAAGGAAACTGGAGAAGACAATCC	TGTACAAGTGGTCCACTTATTTCAATT
ICAM‐1	CACAAGGGCTGTCACTGTTCA	CCCTAGTCGGAAGATCGAAAGTC
PAI‐1	AGCTGGGCATGACTGACATCT	GCTGCTCTTGGTCGGAAAGA
Adiponectin	CAAGGCCGTTCTCTTCACCT	CCCCATACACTTGGAGCCAG
CD4	ACCCTAAGGTCTCTGACCCC	TAGGCTGTGCGTGGAGAAAG
CD8	CACTAGGCTCCAGGTTTCCG	CGCAGCACTTCGCATGTTAG
CD11c	CTGTCATCAGCAGCCACGA	ACTGTCCACACCGTTTCTCC
F4/80	GCCATAGCCACCTTCCTGTT	ATAGCGCAAGCTGTCTGGTT
FoxP3	CTCCAGTACAGCCGGACAC	GGTTGGGCATCAGGTTCTTG
eNOS	AGGCATCACCAGGAAGAAGA	GGCCAGTCTCAGAGCCATAC
iNOS	GGTGAGGGGACTGGACTTTT	CCAACTCTGCTGTTCTCCGT
nNOS	GGACCAGCCAAAGCAGAGAT	GAGCTTTGTGCGATTTGCCA
Endothelin‐1	TTGCTCCTGCTCCTCCTTGAT	TAGACCTAGAAGGGCTTCCTAGT
p22phox	ACCTGACCGCTGTGGTGAA	GTGGAGGACAGCCCGGA
p47phox	ACGCTCACCGAGTACTTCAACA	TCATCGGGCCGCACTTT
UCP‐1	CCGGTGGATGTGGTAAAAAC	CTCCAAGTCGCCTATGTGGT
PPARGC‐1‐*α*	GGGGCACATCTGTTCTTCCA	GAGCTGTTTTCTGGTGCTGC

### Histology assessments

Formalin‐fixed AT samples were processed through paraffin embedment, sectioned at five microns, stained with hematoxylin and eosin for morphometric determinations. Sections were examined using an Olympus BX60 photomicroscope (Olympus, Melville, NY) and photographed at 40× magnification using with a Spot Insight digital camera (Diagnostic Instruments, Inc., Sterling Heights, MI) (Jenkins et al. 2012; Padilla et al. 2013c).

### Functional assessment of isolated aortic rings

A segment of the thoracic aorta, trimmed of AT and connective tissue, was sectioned into 2‐mm rings in cold Krebs. Rings were then mounted on wire feet connected to isometric force transducers and submerged in 20 mL baths containing physiological Krebs solution maintained at 37°C for 1 h to allow for equilibration. Aortic rings were stretched to optimal length which ranged from 130% to 140% of passive diameter. Aortic vasomotor function was investigated with cumulative concentration–response curves of acetylcholine (ACh, 10^−10^ to 10^−4^ mol/L), an endothelium‐dependent dilator and sodium‐nitro‐prusside (SNP, 10^−10^ to 10^−4^ mol/L), an endothelium‐independent dilator. A submaximal concentration of phenylephrine (3e^−7^ mol/L) was used to preconstrict all vessels prior to ACh and SNP relaxation curves. Relaxation at each concentration was measured and expressed as percent maximum relaxation, where 100% is equivalent to loss of all tension developed in response to phenylephrine (Bunker et al. 2010; Padilla et al. 2013c).

### Statistical analysis

The effects of obesity and L‐NAME on body composition and plasma markers were evaluated using 2 × 2 (group × condition) analysis of variances (ANOVAs). Concentration–response curves from vasomotor function experiments were analyzed using 2 × 2 × 7 (group ×condition × concentration) ANOVAs. In addition, 2 ×2 × 3 (group × condition × fat depot) ANOVAs were used to evaluate the effects of obesity, L‐NAME, and fat depot on gene expression of AT samples. A 2 × 2 (group × condition) ANOVAs were used to evaluate the effects of obesity and L‐NAME on gene expression of aortic samples. Simple effects of group, condition, and fat depot were evaluated, and, when appropriate, the Fishers protected least significant difference post hoc was used. All data are presented as mean ± standard error (SE). For all statistical tests, the alpha level was set at 0.05. All statistical analyses were performed with SPSS V21.0 (IBM SPSS, Armonk, NY).

## Results

**Figure 1.**
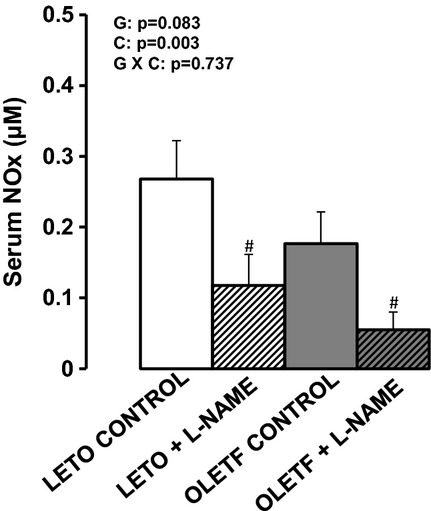
Serum nitrite + nitrate (NOx) levels in LETO and OLETF rats chronically treated without and with L‐NAME. Serum was obtained at 20 weeks (time of sacrifice). Values are expressed as means ± SE. ^#^Difference (*P* < 0.05) from control rats. G, group; C, condition; G × C, group by condition interaction.

Daily L‐NAME intake averaged 71.2 ± 1.3 mg/kg per day in the LETO + L‐NAME group and 67.7 ± 1.6 mg/kg per day in the OLETF + L‐NAME group (between‐group comparison *P* = 0.109). All animals tolerated the L‐NAME treatment well throughout the 4‐week intervention. As expected (McAllister et al. 2008), treatment of L‐NAME resulted in a decrease in serum NOx levels in both groups of rats (Fig. 1).

**Figure 2.**
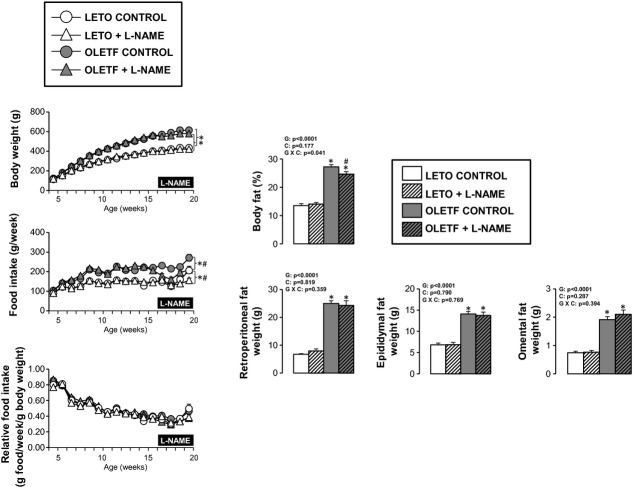
Body composition and food intake in LETO and OLETF rats chronically treated without and with L‐NAME. Values are expressed as means ± SE. Body fat and fat pad weights were obtained at 20 weeks (time of sacrifice). *Difference (*P* < 0.05) from LETO rats; ^#^Difference (*P* < 0.05) from control rats. For body weight and food intake, statistical analysis was performed at 20 weeks. G, group; C, condition; G × C, group by condition interaction.

As shown in Figure 2, OLETF rats were heavier and had a greater percent body fat than LETO rats. L‐NAME treatment produced a small but statistically significant decrease in percent body fat in OLETF, but not LETO, rats. This effect of L‐NAME on the body composition of OLETF rats may be related to decreased food intake induced by L‐NAME (Fig. 2). Retroperitoneal, epididymal, and omental fat pad weights were greater in the OLETF rats than LETO rats and unaffected by L‐NAME treatment. Given that lean mass was unaffected by L‐NAME, from these observations, we deduce that the L‐NAME‐induced reduction in percent body fat of OLETF rats was likely explained by changes in subcutaneous AT; however, total subcutaneous fat mass was not assessed in the present study.

In addition, fasting plasma levels of total cholesterol, high‐density lipoprotein (HDL), nonesterified fatty acids, triglycerides, insulin, glucose, homeostasis model assessment of insulin resistance (HOMA‐IR), and leptin were significantly higher in OLETF rats compared to LETO rats (Table 2). Plasma MCP‐1 levels were significantly lower in OLETF + L‐NAME rats compared to LETO + L‐NAME rats. L‐NAME significantly increased HDL as well as decreased insulin and HOMA‐IR in OLETF rats. These effects were not noted in the LETO rats.

**Table 2.** Fasting plasma characteristics in LETO and OLETF rats chronically treated without and with L‐NAME.

Variable	LETO CONTROL	LETO + L‐NAME	OLETF CONTROL	OLETF + L‐NAME
Total cholesterol, mg/dL	77.0 ± 2.1	80.1 ± 2.0	101.0 ± 3.3*	107.3 ± 3.4*
LDL, mg/dL	41.9 ± 1.4	41.3 ± 2.0	40.0 ± 1.9	40.4 ± 2.5
HDL, mg/dL	27.1 ± 0.6	28.2 ± 0.5	32.4 ± 0.7*	34.4 ± 0.7**
Triglycerides, mg/dL	40.1 ± 2.4	52.9 ± 2.6	142.8 ± 10.0*	162.5 ± 9.0*
NEFA, mmol/L	0.31 ± 0.03	0.32 ± 0.04	0.61 ± 0.04*	0.61 ± 0.03*
Insulin, ng/mL	8.1 ± 1.2	10.1 ± 1.6	32.0 ± 3.8*	22.4 ± 3.1**
Glucose, mg/dL	186.0 ± 13.7	178.1 ± 5.7	309.8 ± 23.5*	312.1 ± 19.7*
HOMA‐IR index	3.9 ± 0.8	4.6 ± 0.8	25.2 ± 4.1*	16.9 ± 2.3**
Leptin, ng/mL	5.3 ± 0.9	4.9 ± 0.8	191.9 ± 59.9*	205.1 ± 54.7*
MCP‐1, pg/mL	282.8 ± 57.6	322.6 ± 45.8	209.1 ± 26.8	167.3 ± 8.9*
TNF‐*α*, pg/mL	5.5 ± 0.5	6.0 ± 0.3	6.8 ± 0.8	5.6 ± 0.2
IL‐6, pg/mL	147.0 ± 39.4	196.1 ± 33.5	190.9 ± 58.4	174.3 ± 37.0

Values are expressed as means ± SE. LDL, low‐density lipoprotein; HDL, high‐density lipoprotein; NEFA, nonesterified fatty acids; HOMA‐IR, homeostasis model assessment of insulin resistance; MCP‐1, monocyte chemotactic protein‐1; TNF‐*α*, tumor necrosis factor alpha; IL‐6, interleukin 6.

* Difference (*P* < 0.05) from LETO rats.

* Difference (*P* < 0.05) from control rats.

**Figure 3.**
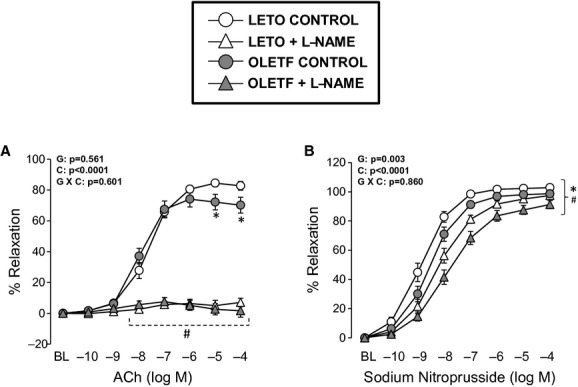
Vasomotor function of thoracic aortic rings in LETO and OLETF rats chronically treated without and with L‐NAME. Values are expressed as means ± SE. (A) *Difference (*P* < 0.05) from LETO rats; ^#^Difference (*P* < 0.05) from control rats. (B) *Difference (*P* < 0.05) from LETO rats under control conditions at dose −9 log M and from LETO rats under L‐NAME conditions at doses −8 to −4 log M. ^#^Difference (*P* < 0.05) from control LETO rats at doses −10 to −4 log M and from control OLETF rats at doses −9 to −4 log M. G, group; C, condition; G × C, group by condition interaction.

As illustrated in Figure 3, ACh‐mediated relaxation of the aorta was blunted in OLETF rats compared to LETO rats at the highest doses of ACh. Aortas from LETO and OLETF rats treated with L‐NAME did not respond to ACh. Dose–response curves to SNP were shifted to the right in OLETF rats (Log EC_50_ = −8.55 ± 0.13) compared to LETO rats (Log EC_50_ = −8.96 ± 0.16, *P* < 0.05). L‐NAME treatment further shifted the SNP dose–response curve to the right in both LETO (Log EC_50_ L‐NAME = −8.31 ± 0.17) and OLETF (Log EC_50_ L‐NAME = −7.92 ± 0.14) rats (both *P* < 0.05).

**Figure 4.**
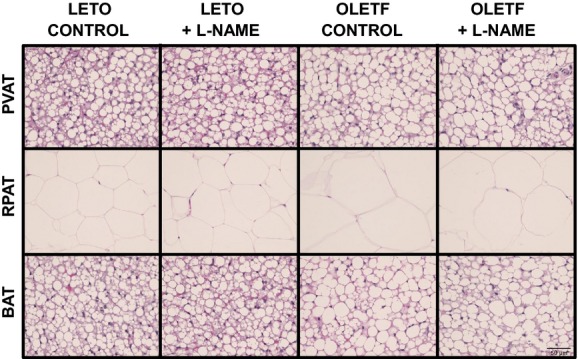
Representative histology photographs (40× magnification) of perivascular (PVAT), retroperitoneal (RPAT) and brown (BAT) ATs in LETO and OLETF rats chronically treated without and with L‐NAME. Samples were stained with hematoxylin and eosin.

Figure 4 illustrates representative histological photographs of perivascular AT, retroperitoneal AT, and brown AT. As shown, obesity was associated with increased lipid deposition in perivascular and brown AT as well as increased adipocyte size in retroperitoneal AT. No effects of L‐NAME treatment on these parameters were observed in any of the AT depots. Consistent with our previous report (Padilla et al. 2013c), a clear structural similarity between thoracic perivascular AT and subscapular brown AT can also be appreciated.

**Figure 5.**
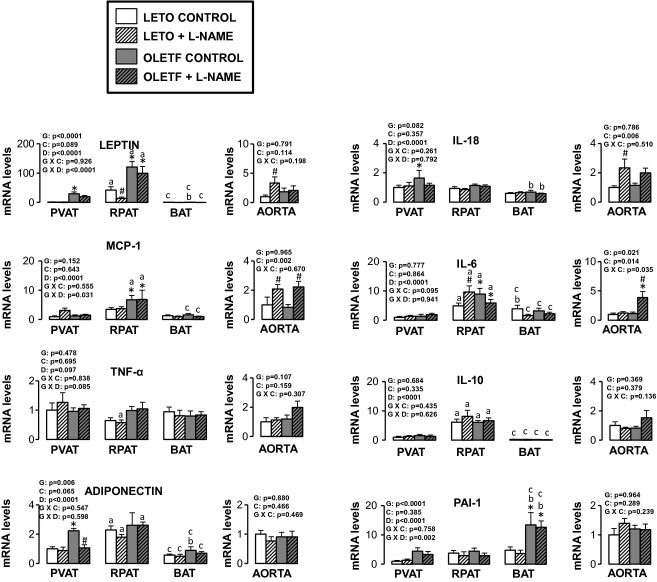
Expression of adipokines and inflammation‐related genes in AT and aorta of LETO and OLETF rats chronically treated without and with L‐NAME. Values are fold difference in mRNA and expressed as means ± SE. PVAT in the LETO control group of rats is used as the reference tissue and set at 1 for all AT comparisons. For aorta comparisons, the LETO control is used as the reference group and set at 1. *Difference (*P* < 0.05) from LETO rats; ^#^Difference (*P* < 0.05) from control rats; ^a^Difference (*P* < 0.05) between PVAT and RPAT; ^b^Difference (*P* < 0.05) between PVAT and BAT; ^c^Difference (*P* < 0.05) between RPAT and BAT. G, group; C, condition; D, fat depot.

**Figure 6.**
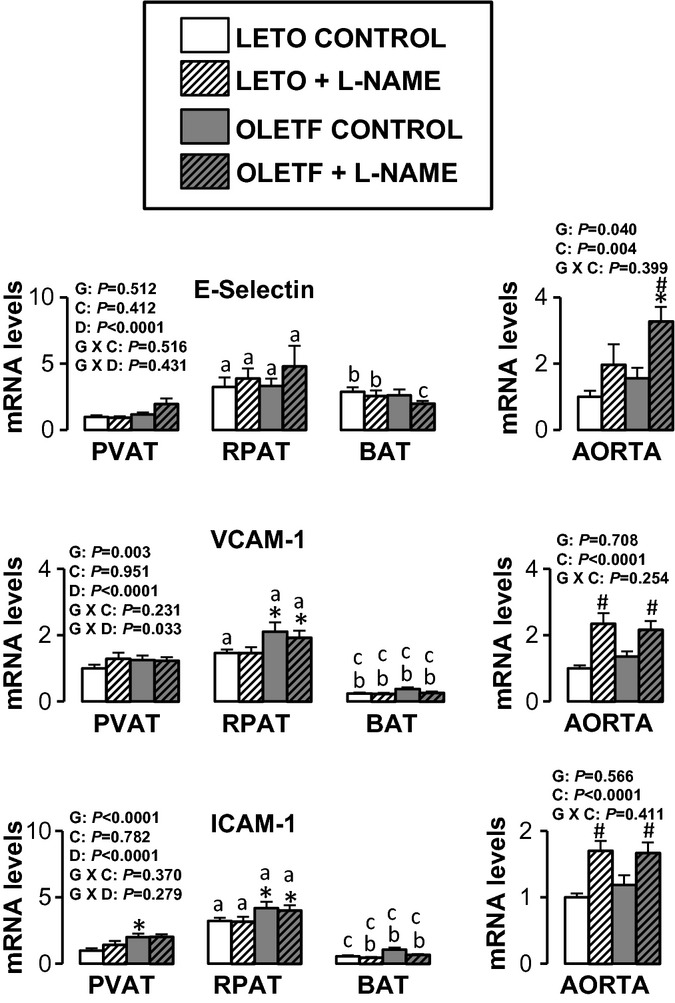
Expression of adhesion molecule‐related genes in AT and aorta of LETO and OLETF rats chronically treated without and with L‐NAME. Values are fold difference in mRNA and expressed as means ± SE. PVAT in the LETO control group of rats is used as the reference tissue and set at 1 for all AT comparisons. For aorta comparisons, the LETO control is used as the reference group and set at 1. *Difference (*P* < 0.05) from LETO rats; ^#^Difference (*P* < 0.05) from control rats; ^a^Difference (*P* < 0.05) between PVAT and RPAT; ^b^Difference (*P* < 0.05) between PVAT and BAT; ^c^Difference (*P* < 0.05) between RPAT and BAT. G, group; C, condition; D, fat depot.

**Figure 7.**
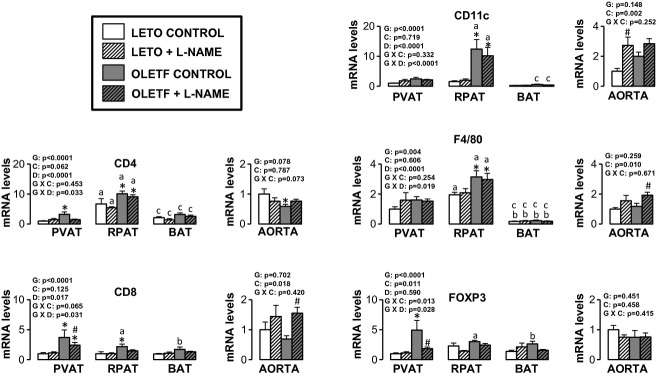
Expression of immune cell‐related genes in AT and aorta of LETO and OLETF rats chronically treated without and with L‐NAME. Values are fold difference in mRNA and expressed as means ± SE. PVAT in the LETO control group of rats is used as the reference tissue and set at 1 for all AT comparisons. For aorta comparisons, the LETO control is used as the reference group and set at 1. *Difference (*P* < 0.05) from LETO rats; ^#^Difference (*P* < 0.05) from control rats; ^a^Difference (*P* < 0.05) between PVAT and RPAT; ^b^Difference (*P* < 0.05) between PVAT and BAT; ^c^Difference (*P* < 0.05) between RPAT and BAT. G, group; C, condition; D, fat depot.

**Figure 8.**
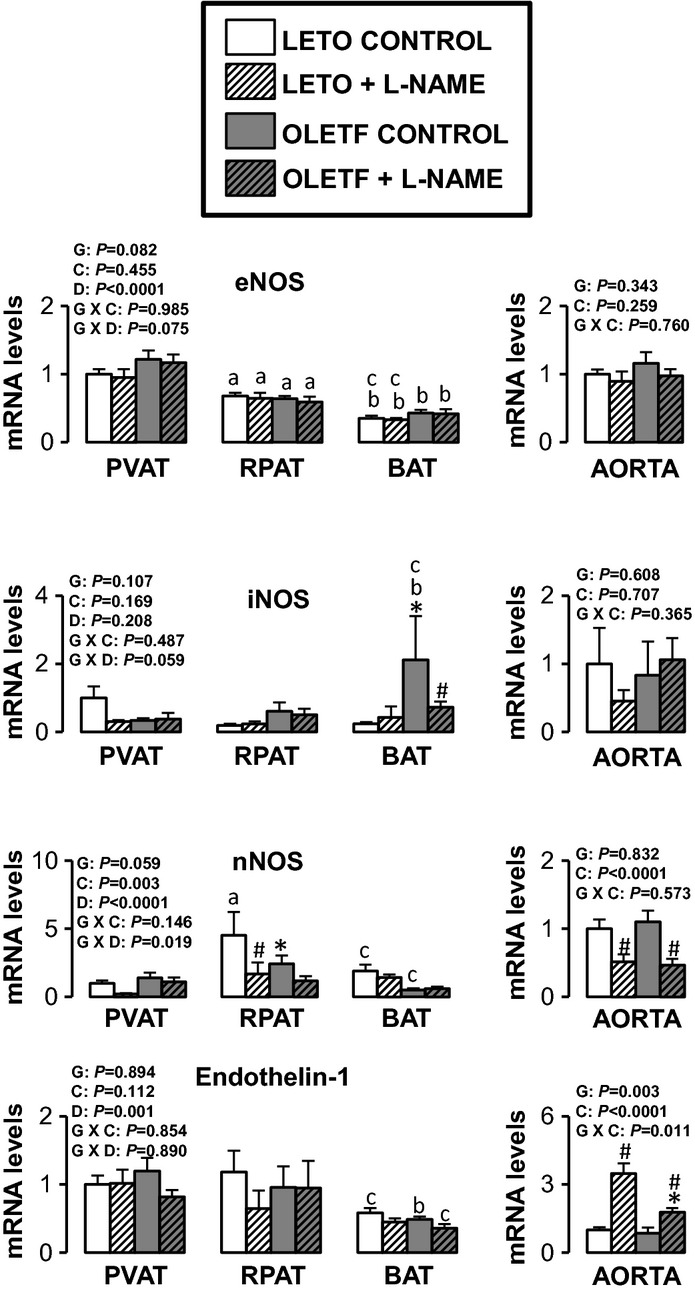
Expression of nitric oxide synthase isoforms and endothelin‐1 genes in AT and aorta of LETO and OLETF rats chronically treated without and with L‐NAME. Values are fold difference in mRNA and expressed as means ± SE. PVAT in the LETO control group of rats is used as the reference tissue and set at 1 for all AT comparisons. For aorta comparisons, the LETO control is used as the reference group and set at 1. *Difference (*P* < 0.05) from LETO rats; ^#^Difference (*P* < 0.05) from control rats; ^a^Difference (*P* < 0.05) between PVAT and RPAT; ^b^Difference (*P* < 0.05) between PVAT and BAT; ^c^Difference (*P* < 0.05) between RPAT and BAT. G, group; C, condition; D, fat depot.

**Figure 9.**
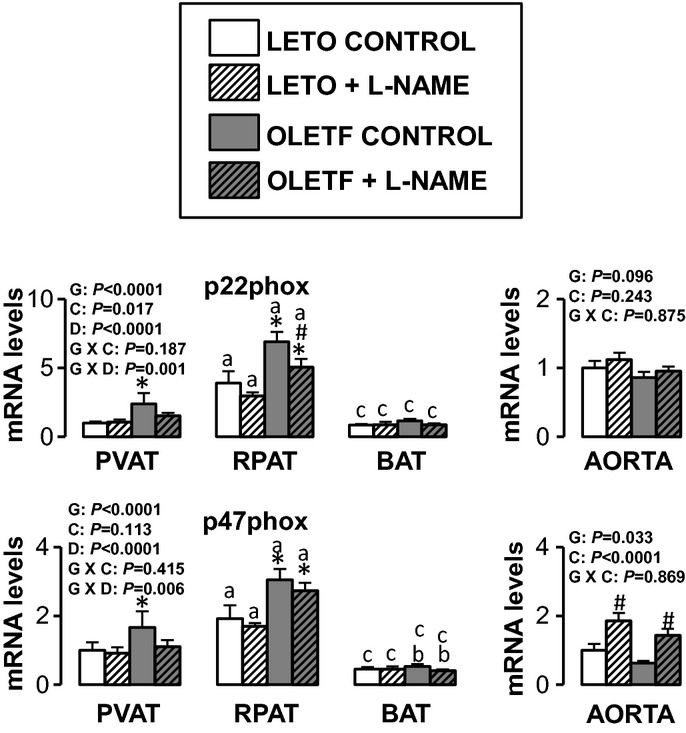
Expression of nitric oxide synthase isoforms and endothelin‐1 genes in AT and aorta of LETO and OLETF rats chronically treated without and with L‐NAME. Values are fold difference in mRNA and expressed as means ± SE. PVAT in the LETO control group of rats is used as the reference tissue and set at 1 for all AT comparisons. For aorta comparisons, the LETO control is used as the reference group and set at 1. *Difference (*P* < 0.05) from LETO rats; ^#^Difference (*P* < 0.05) from control rats; ^a^Difference (*P* < 0.05) between PVAT and RPAT; ^b^Difference (*P* < 0.05) between PVAT and BAT; ^c^Difference (*P* < 0.05) between RPAT and BAT. G, group; C, condition; D, fat depot.

**Figure 10.**
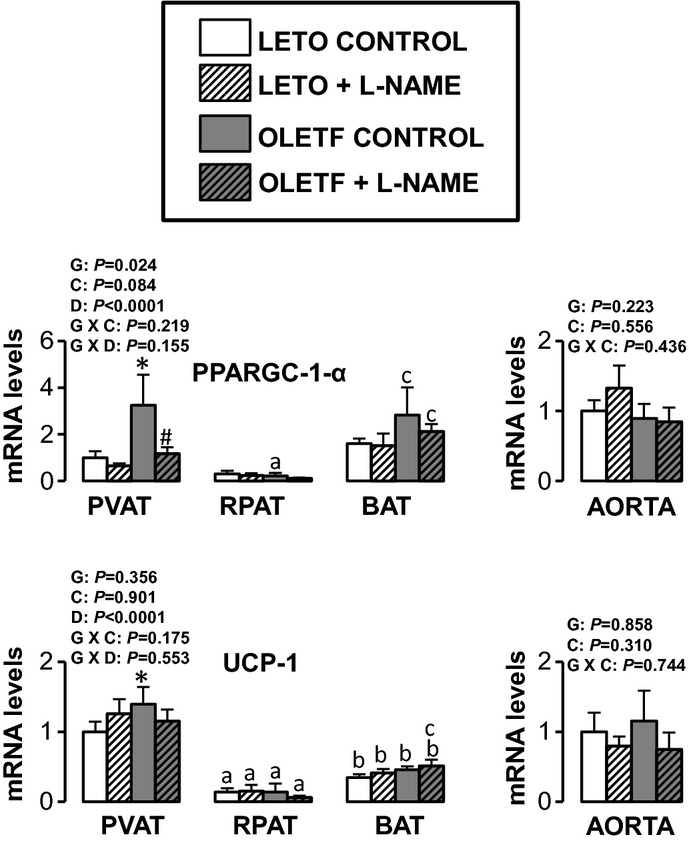
Expression of mitochondria‐related genes in AT and aorta of LETO and OLETF rats chronically treated without and with L‐NAME. Values are fold difference in mRNA and expressed as means ± SE. PVAT in the LETO control group of rats is used as the reference tissue and set at 1 for all AT comparisons. For aorta comparisons, the LETO control is used as the reference group and set at 1. *Difference (*P* < 0.05) from LETO rats; ^#^Difference (*P* < 0.05) from control rats; ^a^Difference (*P* < 0.05) between PVAT and RPAT; ^b^Difference (*P* < 0.05) between PVAT and BAT; ^c^Difference (*P* < 0.05) between RPAT and BAT. G, group; C, condition; D, fat depot; UCP, uncoupling protein.

**Figure 11.**
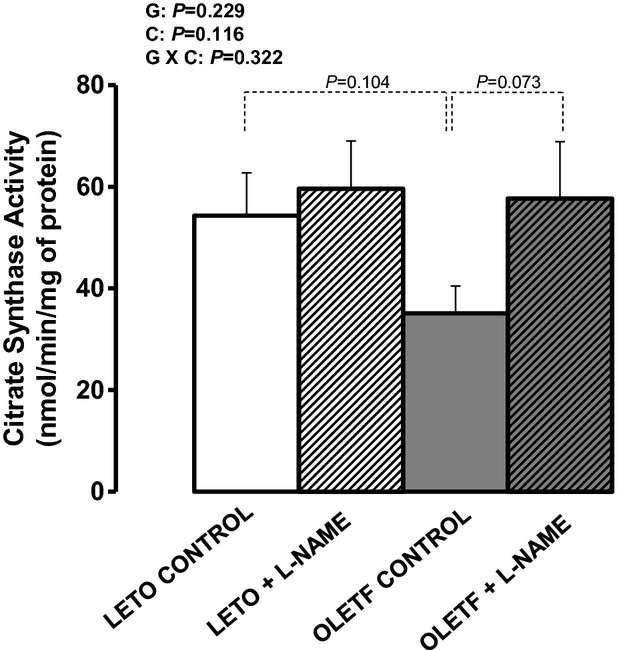
Citrate synthase activity, a marker of mitochondrial content, in retroperitoneal AT of LETO and OLETF rats chronically treated without and with L‐NAME. Values are expressed as means ± SE. G, group; C, condition; G × C, group by condition interaction.

Figures 5–10 summarize the results on AT and vascular gene expression. Specifically, adipokines and inflammation‐related genes are presented in Figure 5, adhesion molecule‐related genes are presented in Figure 6, immune cell‐related genes are presented in Figure 7, NO isoforms and endothelin‐1 are presented in Figure 8, NADPH oxidase‐related genes are presented in Figure 9, and mitochondria‐related genes are presented in Figure 10. For AT, there was a significant main effect of group for leptin, vascular cell adhesion molecule (VCAM)‐1, intracellular adhesion molecule (ICAM)‐1, plasminogen activator inhibitor (PAI)‐1, adiponectin, CD4, CD8, CD11c, F4/80, FoxP3, p22phox, p47phox, and peroxisome proliferator activated receptor gamma, coactivator (PPARGC)‐1‐*α* mRNA (all increased in OLETF relative to LETO rats). A significant main effect of L‐NAME treatment was only observed for FoxP3, nNOS, and p22phox mRNA (all three decreased in L‐NAME treated rats relative to control rats). A significant main effect of AT depot was observed for all mRNAs examined except for TNF‐*α* (*P* = 0.097), FoxP3 (*P* = 0.590), and iNOS mRNA (*P* = 0.208). A significant group by condition interaction was only observed for FoxP3 mRNA. For clarity and as an example, a statistical interaction occurs when differences between levels (e.g., control vs. L‐NAME) within one group (e.g., LETO) are not the same as the differences between levels in another group (e.g., OLETF). A significant group by AT depot interaction was observed for leptin, MCP‐1, VCAM‐1, PAI‐1, CD4, CD8, CD11c, F4/80, FoxP3, nNOS, p22phox, and p47hpox mRNA. A significant group by condition by AT depot interaction was only observed for IL‐6 mRNA. Figure 11 illustrates the effects of obesity and L‐NAME treatment on citrate synthase activity in the retroperitoneal AT. Although not statistically significant, relative to LETO rats, retroperitoneal AT from OLETF rats appeared to have reduced levels of citrate synthase activity by 35% (*P* = 0.104), an effect that was normalized with L‐NAME treatment (*P* = 0.073).

For aortic samples, there was a significant main effect of group for five mRNAs. IL‐6 and E‐selectin mRNA levels were higher in OLETF relative to LETO rats, and endothlein‐1, GRP78, and p47phox mRNA levels were lower in OLETF relative to LETO rats. A significant main effect of L‐NAME treatment was observed for MCP‐1, IL‐6, IL‐18, E‐selectin, VCAM‐1, ICAM‐1, CD8, CD11c, F4/80, endothelin‐1, and p47phox mRNA (all increased in L‐NAME treated rats relative to control rats), as well as for nNOS mRNA, which decreased in L‐NAME treated rats relative to control rats. A significant group by condition interaction was only observed for IL‐6 and endothelin‐1 mRNA. Significant main effects of group, condition, and fat depot on gene expression are depicted in the figures (Figs. 5–10).

## Discussion

With increasing evidence that AT contributes to the pathogenesis of metabolic and cardiovascular diseases through the local and systemic secretion of proinflammatory cytokines (Mazurek et al. 2003; Lau et al. 2005; Ronti et al. 2006; Cheng et al. 2008; Gorter et al. 2008; Chatterjee et al. 2009; Greif et al. 2009; Anderson et al. 2010; Surmi and Hasty 2010; Li et al. 2011; Payne et al. 2012a,b; Szasz and Webb 2012; Stohr and Federici 2013), a deeper understanding of the mechanisms responsible for the phenotypic modulation of AT is needed. The main purpose of this study was to test the hypothesis that a decrease in bioavailability of NO would result in increased AT inflammation. This was accomplished by examining the extent to which chronic inhibition of NOS, in the presence or absence of obesity, altered inflammatory gene expression in retroperitoneal white AT, subscapular brown AT, periaortic AT, and its contiguous aorta free of perivascular AT. Contrary to our hypothesis, we found that expression of inflammatory genes and markers of immune cell infiltration in all AT depots examined were, by and large, unaltered with chronic administration of L‐NAME in both lean and obese rats. This was in contrast with the observation that L‐NAME produced a significant upregulation of inflammatory and proatherogenic genes in the aorta. Collectively, these findings suggest that the impact of systemic NOS inhibition on inflammatory gene expression is greater in the vascular wall relative to its surrounding perivascular AT, or visceral white and subscapular brown AT depots.

Our finding that NOS inhibition generally did not evoke an increase in inflammatory gene expression in AT was somewhat surprising in light of previous research. Using the eNOS knockout mouse model, Handa et al. (2011) demonstrated that reduced eNOS‐derived NO signaling is sufficient to induce expression of proinflammatory cytokines and markers of immune cell infiltration in visceral white AT. Likewise, using an eNOS overexpressed mouse model, Sansbury et al. (2012) recently showed that increased eNOS activity prevents the obesogenic effects of high‐fat diet, in part, by stimulating mitochondrial biogenesis and activity in visceral white AT, thus resulting in a decreased adipocyte size.

A possible explanation of the disparity of findings between these studies and our study may be related to the differences in techniques employed to modulate NO signaling (i.e., eNOS knockout/overexpressed rodent models vs. chronic administration of L‐NAME in our study). L‐NAME acts as a competitive inhibitor of NOS due to its structural similarity to l‐arginine, the substrate of NOS, thus inhibiting all NOS isoforms. Our findings, taken together with data from others using NOS transgenic mouse models (Perreault and Marette 2001; Becerril et al. 2010; Handa et al. 2011; Sansbury et al. 2012), suggest that the source of NO (eNOS, iNOS, or nNOS) being modulated may be a determinant of the effects of altered NOS activity on the inflammatory response. Both eNOS and nNOS produce NO in relatively low amounts, whereas iNOS can synthesize remarkably large amounts of NO (Lincoln et al. 1997; Stuehr 1997; Enkhbaatar et al. 2003; Ichinose et al. 2003). Current evidence indicates that low amounts of NO are beneficial while the large quantities of NO produced by iNOS can be harmful (Laroux et al. 2001; Thomas et al. 2008). Specifically, it appears that NO derived from eNOS is a key signaling molecule in maintaining a healthy, anti‐inflammatory AT phenotype (Handa et al. 2011), whereas a reduction in NO derived from iNOS may result in reduced both adipocyte size and inflammation. For example, evidence from iNOS knockout mouse studies indicates that ablation of the iNOS gene protects against diet‐induced obesity and insulin resistance (Perreault and Marette 2001), and in AT, increases expression of mitochondria‐related proteins, and reduces expression of inflammatory cytokines including leptin (Becerril et al. 2010). Hence, given these contrasting roles of eNOS versus iNOS in modulating AT phenotype, the overall net result when inhibiting all NOS isoforms with L‐NAME may be no effect as we indeed largely report in our present study. An alternative explanation could be that the dose of L‐NAME at the AT level was insufficient to effectively inhibit NOS isoforms and produce robust genomic effects. Interestingly, there seems to be a downward trend in inflammatory markers in the AT from OLETF L‐NAME treated rats versus OLETF controls (e.g., IL‐6, CD4, CD8, CD11C, FoxP3). We speculate this may be evidence of inhibition of the overproduction of NO derived from iNOS in the AT of obese rats. Along these lines, we also observed that L‐NAME treatment slightly increased citrate synthase activity, a marker of mitochondrial content, in the retroperitoneal AT of OLETF rats, an effect that would be expected to result from iNOS inhibition (Becerril et al. 2010) and not eNOS inhibition (Sansbury et al. 2012). Indeed, current evidence suggests that eNOS‐derived NO is an important signal for mitochondrial biogenesis in visceral AT (Sansbury et al. 2012).

While NOS inhibition did not produce an effect on AT mRNA levels with the exception of a few genes, we did observe enlargement of adipocyte size and upregulation of inflammatory genes with obesity in the OLETF rat across all AT depots as well as marked differences in gene expression among fat pads. In particular, our data support the idea that perivascular AT surrounding the thoracic aorta has some phenotypic similarities, both morphologically and at the transcriptional level, with brown AT, thus corroborating our recent findings (Padilla et al. 2013c). Importantly, in addition to providing evidence of phenotypic divergence among AT depots, here we show that the effects of hyperphagia‐induced obesity on AT gene expression (leptin, MCP‐1, VCAM‐1, PAI‐1, CD4, CD8, CD11c, F4/80, FoxP3, nNOS, p22phox, and p47hpox mRNA) appear to be heterogeneous across fat pads.

The overall absence of an L‐NAME effect on the phenotype of aortic perivascular AT, and other fat depots examined, is in clear contrast with the NOS inhibition‐induced upregulation of inflammatory genes and markers of immune cell infiltration in the contiguous aortic wall. Our data support earlier research demonstrating the atheroprotective role of vascular NO. For example, it has been shown that inhibition of NOS with L‐NAME produces atherosclerotic lesions in the aorta of hypercholesterolemic rabbits (Cayatte et al. 1994), increases expression of prooxidant and inflammatory genes in the aorta of normal rats (Gomez‐Guzman et al. 2011), and increases leukocyte rolling and adhesion in the human microvasculature (Hossain et al. 2012). In addition, there is evidence that mice with targeted disruption of the eNOS gene exhibit abnormal vascular remodeling in response to external carotid artery ligation (Rudic et al. 1998), and mice with eNOS/apoE double knockout exhibit accelerated atherosclerosis, aortic aneurysm formation, and ischemic heart disease (Kuhlencordt et al. 2001, 2009). One of the unique aspects of our L‐NAME study, relative to previous research, is the inclusion of lean and obese rats. We observed an obesity‐associated impairment in ACh and SNP‐mediated relaxation in aortic rings. In addition, aortas from L‐NAME‐treated rats, both lean and obese, exhibited complete abrogation of ACh‐mediated relaxation. Furthermore, L‐NAME treatment reduced SNP‐mediated vascular responsiveness and the extent of this effect was similar in both lean and obese rats. The observation that L‐NAME treatment did not abolish between‐group differences in SNP‐mediated relaxation, suggest that the effect of obesity on vascular responsiveness to NO may not be due to differences in NOS activity.

Of interest, while we observed an effect of obesity on aortic vasomotor function, these effects were not associated with changes in vascular gene expression. Indeed, overall, we did not detect significant differences in aortic mRNA levels between LETO and OLETF rats in the absence of L‐NAME. Furthermore, although the vascular effects of NOS inhibition were largely uniform between groups of rats, there were a few exceptions where NOS inhibition unmasked the obesity effect on vascular inflammatory gene expression. Specifically, we noted that induction of E‐selectin and IL‐6 mRNAs with NOS inhibition was apparent in the obese but not the lean rats. The same was true for other genes including TNF‐*α* and IL‐10; however, these effects did not reach statistical significance.

Limitations of the present investigation should be considered. First, our study did not establish whether the reported changes in AT mRNA levels are attributable to alterations in the phenotype of adipocytes and/or resident immune cells within the AT. Similarly, because we studied mRNA levels from whole artery homogenates, it is unknown whether differences in aortic gene expression reported in this study are originating from the endothelium, smooth muscle, or adventitia. Examination of the impact of NOS inhibition and obesity on vascular gene expression with separation of cell populations should be a priority in future studies. Second, all our vasomotor function experiments in the aorta were performed in the absence of perivascular AT. Future studies are needed to determine if inhibition of NOS in perivascular AT alters vasomotor reactivity. Third, our group and others (Lloyd et al. 2001; Gomez‐Guzman et al. 2011) have previously established that daily consumption of L‐NAME in drinking water increases mean arterial pressure in rats. In this regard, because there is extensive evidence that increased blood pressure is a proatherogenic stimulus to the vasculature (Padilla et al. 2013a), at this time we cannot establish the extent to which the effects of L‐NAME on aortic gene expression reported herein are attributable to an increased blood pressure versus primarily the direct result of vascular NOS inhibition. This is an important limitation to the present study and further research is necessary to tease out the contribution of increased blood pressure versus local removal of NO signaling in modulating vascular gene expression. A potential approach for excluding hypertension‐induced changes would be to administer an antihypertensive therapy to L‐NAME‐treated rats.

In summary, this was the first study to evaluate the effects of systemic NOS inhibition on AT gene expression across different fat pads in lean and obese rats. We provide evidence that expression of inflammatory genes and markers of immune cell infiltration in AT were largely unaltered with chronic administration of L‐NAME. This observation is in contrast with the finding that L‐NAME caused an overall upregulation of inflammatory genes in the aorta. Taken together, these data suggest that systemic NOS inhibition alters transcriptional regulation of proinflammatory genes to a greater extent in the aortic wall compared to its surrounding perivascular AT, as well as relative to visceral white and subscapular brown AT depots.

## Conflict of Interest

None declared.
